# Influence of electronic cigarette vapour on the *in vitro* chromatic stability of different nanohybrid composite resins

**DOI:** 10.2340/biid.v12.44849

**Published:** 2025-10-13

**Authors:** Adriana Abanto-Vásquez, Teresa Ulloa-Cueva, Carol Portales-Carbonel, Yuri Curo-Valdivia

**Affiliations:** Stomatology Study Program, Faculty of Human Medicine, Universidad Privada Antenor Orrego, Trujillo, Peru

**Keywords:** smoking, vaping, electronic nicotine delivery systems, composite resins, oral health, dental aesthetics

## Abstract

**Objective:**

The purpose of this study was to compare the chromatic differences (ΔE) of three nanohybrid composite resins (Filtek™ Z350 XT, FORMA™ and Palfique LX5) after increasing exposures (0, 250, 500, 750, 1,000, 1,250 and 1,500 puffs) to e-cigarette vapours.

**Materials and methods:**

The study was conducted with an *in vitro* experimental design, in which a total of 66 resin discs measuring 10 mm × 2 mm were used and distributed as follows: 22 discs of each brand of resin tested. After 24 hours storage in water at 37°C and 500 cycles of thermal cycling (5°C–55°C), they were exposed to the vapour of a ‘Vuse’ brand electronic cigarette dispensed in an artificial vacuum system, graded into levels consisting of six puff each. Colour parameters were measured according to the CIELab system with a Konica Minolta CR-400 colorimeter before (baseline) and after each cycle of puffs, and the difference in colour (ΔE) was calculated. For statistical analysis, the mixed analysis of variance (ANOVA) test and the post hoc test of Games‑Howell, was used, with a confidence level of 95%.

**Results:**

Filtek™ Z350 XT resin had an average ΔE value of 3.239 ± 1.30; FORMA™ showed an ΔE value of 4.737 ± 1.672, and Palfique LX5 had a mean ΔE of 1.614 ± 0.867. Significant differences were found between resin brands (*p* < 0.001), while increasing puffs did not significantly influence ΔE within each resin (*p* > 0.05).

**Conclusion:**

Chromatic stability of composites after exposure to e-cigarette vapours varied depending on the material brand, with FORMA™ resin showing clinically unacceptable chromatic differences, whereas Filtek™ Z350 XT and Palfique LX5 resins showed clinically acceptable chromatic differences, with the latter showing greater resistance to colour change and therefore greater chromatic stability. These findings serve as a guide in decision-making relative to the selection of restorative materials indicated for patients with vaping habits.

## Introduction

The use of electronic cigarettes has increased considerably in recent years, arousing the dental community’s interest considering the potential effects of vapour on restorative materials [1]. Unlike conventional tobacco smoke, these devices release an aerosol composed of nicotine, glycerine, propylene glycol and flavourings, which can affect various properties of dental materials [2]. These components can specifically interact with the surface of restorative materials and enamel, affecting chromatic stability and, potentially, surface integrity; they may also cause an increase in bacterial adhesion and biofilm formation, which could have additional implications for oral health [3–6].

Reports of recent evidence have indicated that exposure to e-cigarette vapour produces significant changes in the chromatic stability of lithium disilicate veneers, especially when flavoured liquids and high nicotine concentrations are used [7]. Moreover, it has been observed that some ceramic materials, such as layered zirconia, showed colour changes above the clinically acceptable threshold after multiple exposures to cigarette vapour [4]. This is why it is mentioned that the flavour and nicotine concentration of electronic cigarette liquids could significantly influence the colour alteration of restorative dental materials [8].

There has been a substantial percentage increase in the use of e-cigarettes in recent years, both among adolescents and young adults, with relative increases exceeding 50% in several countries and among age groups around the world [9–11]. In this context, composite resins, which are widely used in restorative dentistry due to their aesthetic and adhesive properties, constitute approximately 60% of restorative treatments worldwide, and are the main treatment for dental caries [12]. This raises the question of whether resin chromatic stability can be affected by external factors, such as the consumption of certain foods, beverages, or products, including electronic cigarettes. However, scientific evidence on the effect of e-cigarettes on composite resins continues to be limited.

Some studies show that colour change of resins is due both to surface adsorption and to absorption into the organic matrix, and that the accumulation of these pigmenting agents can reach a plateau where additional exposures produce small changes. This could explain how pigmenting agents can cause chromatic alterations by superficial and intrinsic mechanisms [13–15]. Since these resins are widely used for their aesthetic appearance and adaptability, it is essential to investigate the effects of the aerosol generated by e-cigarette vapour under controlled conditions, in order to obtain an approximation of the behaviour of these components on the various dental restorations present in the population at present.

Nanohybrid resins combine particles of different sizes to provide good polishability, aesthetics, and strength, which makes their use common for anterior and posterior restorations. Because of their balance between mechanical properties and appearance, evaluating various commercial brands is of interest to compare chromatic stability against different cycles of vapour puffs [16, 17]. We hypothesised that exposure to electronic cigarette vapour alters the chromatic stability of composite resins used in dental restorations *in vitro*. Therefore, the main objective of this study was to conduct an *in vitro* comparison of the chromatic stability of three brands of nanohybrid composite resins, exposed to different numbers of puffs of electronic cigarette vapour.

## Materials and methods

The present *in vitro* study with an experimental design was conducted in the laboratory of the Agricultural Engineering and Agro-export study programme of the Universidad Privada Antenor Orrego in Trujillo, Peru, with the approval of the Ethics Committee of the Universidad Privada Antenor Orrego No. 000459-2025-UPAO.

### Materials selected

Three following brands of nanohybrid composite resins commonly used in clinical dental practice were selected: (Filtek™ Z350 XT (3M™), FORMA™ (ULTRADENT®) and Palfique LX5 (TOKUYAMA®). Shade A2 was selected as the reference shade because it is frequently used in studies of chromatic stability of composite resins; this choice enabled a more accurate assessment of colour changes and minimised the influence of the initial shade on result variability [18]. Table 1 shows the specific composition of each resin.

**Table 1:** Characteristics of nanohybrid composite resins used in research.

N°	Brand	Composition	Manufacturer	Lot number
**1.**	**Filtek™ Z350 XT**	Bis-GMA, Bis-EMA, UDMA, TEGDMA. Contains a 20 nm non-agglomerated/non-aggregated nanosilica filler and a loosely bonded zirconium/silica nanocluster consisting of agglomerates of 5–20 nm zirconium/silica primary particles The particle size of the aggregate ranges from 0.6 to 1.4 microns. The particle size of the aggregate ranges from 0.6 to 1.4 microns.	3M™	A: 11214638
				B: 10946988
**2.**	**FORMA™**	Silicon dioxide, chemically prepared ≥ 0 – ≤ 25%, TEGDMA, UDMA, Zirconium dioxide ≥ 0 – ≤ 10%, Chromium iron oxide ≥ 0 – ≤ 10%, Titanium dioxide.	ULTRADENT®	A: D0M98
				B: D0Q9I
**3.**	**Palfique LX5**	Matrix of Bis-GMA and TEGDMA monomers, and a high content of inorganic filler (82% by weight or 71% by volume) made of silica and zirconium dioxide.	TOKUYAMA®	A: 304E03
				B: 284E63

### Sample size calculation

The sample size calculation for the comparison of multiple groups was determined using G-power software (G*Power 3.1.9.7, Dusseldorf, Germany). The requirements of a 95% confidence interval (CI) of the normal distribution coefficient (α/2 = 0.05 *Z* = 1.96) and a statistical power of 90% (β = 0.10; *Z* = 1.282) were assumed. Moreover, a previous study conducted by Alrabeah et al. [4] was taken into account considering an effect size of 0.45. A total of 22 (*N* = 22) samples were obtained for each group, of resin, equalling a total of 66 (*N* = 66) samples across all test groups.

### Sample preparation

A stainless steel mould with an internal diameter of 10 mm and depth of 2 mm was used to make the resin discs. The composite resins were put into the stainless steel mould to form discs of the aforementioned size. Each of the samples were then photocured with a VALO™ LED light , at a distance of 1 mm from the sample, at an output power of 1,470 mW/cm^2^, for 40 seconds [12]. The samples were polished with the Jiffy™ kit (ULTRADENT®), in accordance with the manufacturer’s instructions, starting with the coarsest grains to the finest grains (green, yellow and white), for 15 seconds each using low speed.

### Thermal cycling process

To age the samples of all groups, they were previously stored in distilled water at 37°C for a period of 24 hours. After this preparatory phase, the specimens were subjected to 500 cycles of thermal cycling, alternating between temperatures of 5°C and 55°C, with a stay of 60 seconds in each cycle (30 seconds at 5°C and 30 seconds at 55°C). The number of cycles used in this study corresponded to the minimum recommended in laboratory protocols for simulating biomaterial ageing and has been employed in several studies to assess chromatic stability [19].

### Simultaneous exposure to puffs of e-cigarette vapour

**Figure 1:**
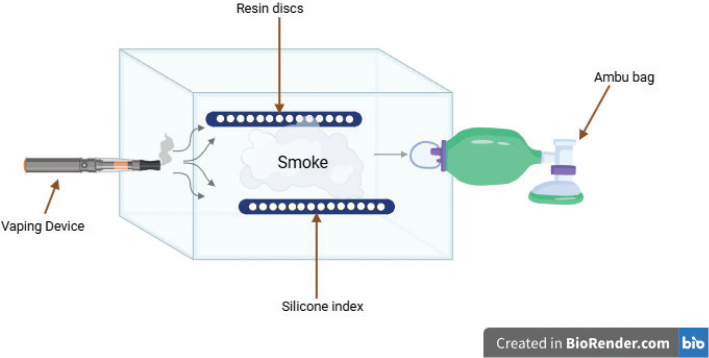
Schematic diagram of the vacuum system with an artificial lung that was used for submitting the nanohybrid composite resin discs to electronic cigarette vapour.

A vacuum system with an artificial lung was used (Figure 1), designed with the primary purpose of directing the smoke into a plastic chamber created specifically for the study. To generate the smoke, an electronic vaping device was used. The ‘Vuse’ model with pre-loaded nicotine, capable of producing up to 1,500 puffs, was specifically selected for its high popularity among consumers [20]. The chemical composition of the aerosol (propylene glycol/vegetable glycerine base with commercial flavourings and nicotine salts) and the puff-generation method were considered comparable to those reported in a similar *in vitro* study, and therefore that study was used as a reference for aerosol concentration and characteristics [4]. The device was adhered to the system with a silicone stick and the mouthpiece was placed inside the chamber, at a distance of 10 cm from the composite resin discs. At the opposite end, a Polyvinyl Chloride (PVC) manual resuscitator (adult model, Greetmed®) was placed and similarly adhered with the aforementioned silicone.

**Figure 2:**
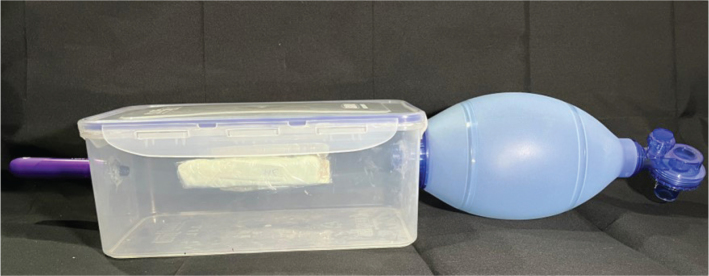
Customised vacuum system with an artificial lung.

After this, the resin discs were simultaneously fixed with heavy condensation silicone (Speedex Putty®). They were aligned horizontally on both sides of the chamber, but oriented vertically rather than lying flat, to minimise unrealistic aerosol residue deposition and ensure more uniform exposure. Prior to each exposure, the electronic device was turned on, and then the manual resuscitator was compressed to allow the aerosol to enter the chamber. By means of the vacuum system the aerosol was drawn from the atomiser into the chamber, allowing uniform exposure of the composite resin discs to the smoke generated (Figure 2).

Exposure was maintained for 40–60 seconds in each cycle, until the smoke dissipated. This procedure was repeated to reach the target number of exposures, distributed in six cycles, organised as follows: 0 (baseline), 250, 500, 750, 1,000, 1,250, and 1,500 puffs. These intervals were selected following the protocol described by Alrabeah et al. [4]. This entire process was applied simultaneously to all groups.

### Measurement of chromatic stability

The principal investigator performed the colour measurements, using a calibrated Konica Minolta CR-400 colorimeter serial No. 8207368, with automated variable sample illumination and a 44 mm objective mask aperture. Since the measurement procedure was performed continuously at the established intervals, the resin discs were not stored in water between exposures, as immersion could have influenced the staining behaviour and confounded the results. This instrument functioned the CIELab colour system, from which the colour difference ‘ΔE’ was obtained, and was calculated by recording an initial sample colour before exposure to electronic cigarette vapour and after exposure for the intervals mentioned above.

Within the CIELab system, there are two chromatic axes, a* (red-green parameter difference) and b* (yellow-blue parameter difference), which are at right angles to each other, and represent the saturation level and the dimensions of hue. A third axis, L*, is perpendicular to the chromatic planes and represents value or lightness. To obtain the CIELab colour difference ‘ΔE’, the calculation was performed using the following formula, as previously described in various studies [4]:

ΔE = [(ΔL*) 2 + (Δa*) 2 + (Δb*) 2]^½^

Where to determine ‘ΔL’, ‘Δa’ and ‘Δb’, the difference between the L*, a*, and b* values before (base colour measurement) and after the exposures (colour measurement after exposures to different number of puffs) is calculated.

Chromatic stability was determined from ‘ΔE’ values and interpreted against the clinical perceptibility and acceptability thresholds described by Alnasser et al. [12], where values below the perceptibility threshold (ΔE < 1) are undetectable to the human eye; values between the perceptibility and acceptability thresholds (1 < ΔE < 3.3) are considered clinically acceptable; and values at or above the acceptability threshold (ΔE ≥ 3.3) are clinically unacceptable.

### Statistical analyses

The data collected were processed and imported into the SPSS v27 programme for analyses. Means and standard deviations of ‘ΔE’ obtained were calculated according to resin type and interval of exposure. In addition, values were compared using a mixed analysis of variance (ANOVA) test, with the between- subject factor being the type of nanohybrid resin and the within-subject factor being the number of e-cigarette vapour puffs. Multiple comparisons were also performed using the Games-Howell post hoc test, since the assumption of homogeneity of variances was not met, considering a significance level of *p* < 0.05 and a CI of 95%.

## Results

In Table 2, the initial parameters are shown, in which the parameter L* showed relatively similar values between the resins. The resin FORMA™ showed the highest luminosity (*L* = 66.983 ± 0.726), followed by Filtek™ Z350 XT (*L* = 63.724 ± 1.44) and Palfique LX5, which showed the lowest value (*L* = 61.210 ± 0.784). In the case of parameter a*, all resins showed negative values, which showed a slight tendency towards greenish tones. The most pronounced value was observed for FORMA™ (*a* = –0.301 ± 0.109), followed by Z350 XT (*a* = –0.17 ± 0.205) and Palfique LX5 (*a* = –0.021 ± 0.223). With respect to parameter b, all resins showed positive values, indicating a tendency towards yellowish tones. The resin FORMA™ had the highest value (*b* = 10.514 ± 0.531), followed by Z350 XT (*b* = 9.122 ± 0.478) and Palfique LX5 (*b* = 8.368 ± 0.562).

**Table 2:** Colour values L, a, and b of composite resins before exposure to e-cigarette vapour puff cycles.

	Filtek™ Z350 XT			FORMA™			Palfique LX5		
	L	a	b	L	a	b	L	a	b
	Mean (SD)	Mean (SD)	Mean (SD)	Mean (SD)	Mean (SD)	Mean (SD)	Mean (SD)	Mean (SD)	Mean (SD)
Baseline parameters	63.724 (1.440)	–0.170 (0.205)	9.122 (0.478)	66.983 (0.726)	–0.301 (0.109)	10.514 (0.531)	61.210 (0.784)	–0.021 (0.223)	8.368 (0.562)

SD: Standard Deviation.

Table 3 shows the parameters of the materials after being exposed to electronic cigarette vapour. In the case of the Filtek™ Z350 XT, the L* value fluctuated slightly between 62.503 ± 1.567 and 66.414 ± 0.796, while the parameter a* remained negative (between –0.963 ± 0.13 and –0.664 ± 0.127) and parameter b* showed values ranging between 9.557 ± 0.311 and 11.293 ± 0.571. In the case of resin FORMA™ resin, L* values ranged between 62.353 ± 1.523 and 62.828 ± 1.662, with a* values varying from –1.092 ± 0.210 to –0.925 ± 0.240 and b* values ranging between 9.278 ± 0.342 to 9.710 ± 0.343. Lastly, Palfique LX5 maintained L* ranging values between 60.103 ± 0.925 and 60.500 ± 1.564, a* values in a range from –0.323 ± 0.274 to –0.122 ± 0.219, and b* values varying from 8.241 ± 0.563 to 8.529 ± 0.616, showing minimal colour changes with increasing numbers of puffs.

**Table 3:** Colour values L, a and b of the composite resins exposed to 250, 500, 750, 1,000, 1,250, and 1,500 puffs of electronic cigarette vapour.

Parameters at post-exposure to electronic cigarette vapour	Filtek™ Z350 XT			FORMA™			Palfique LX5		
	L	a	b	L	A	b	L	a	b
	Mean (SD)	Mean (SD)	Mean (SD)	Mean (SD)	Mean (SD)	Mean (SD)	Mean (SD)	Mean (SD)	Mean (SD)
**250 puffs**	66.414 (0.796)	–0.664 (0.127)	10.855 (0.514)	62.828 (1.662)	–1.092 (0.210)	9.278 (0.342)	60.398 (0.818)	–0.252 (0.204)	8.394 (0.525)
**500 puffs**	66.034 (0.735)	–0.728 (0.128)	11.038 (0.557)	62.354 (1.598)	–0.932 (0.284)	9.625 (0.473)	60.292 (0.814)	–0.122 (0.219)	8.410 (0.529)
**750 puffs**	62.503 (1.567)	–0.934 (0.198)	9.557 (0.311)	62.503 (1.567)	–0.934 (0.198)	9.557 (0.311)	60.459 (0.842)	–0.236 (0.228)	8.241 (0.563)
**1,000 puffs**	65.956 (0.659)	–0.963 (0.130)	11.251 (0.553)	62.435 (1.521)	–0.984 (0.23)	9.629 (0.368)	60.500 (1.564)	–0.323 (0.274)	8.499 (0.745)
**1,250 puffs**	65.880 (0.887)	–0.959 (0.108)	11.293 (0.571)	62.353 (1.523)	–1.000 (0.231)	9.710 (0.343)	60.103 (0.925)	–0.270 (0.234)	8.529 (0.616)
**1,500 puffs**	65.939 (0.672)	–0.843 (0.118)	11.224 (0.581)	62.393 (1.553)	–0.925 (0.240)	9.643 (0.382)	60.227 (0.864)	–0.156 (0.236)	8.414 (0.589)

SD: Standard Deviation.

**Figure 3:**
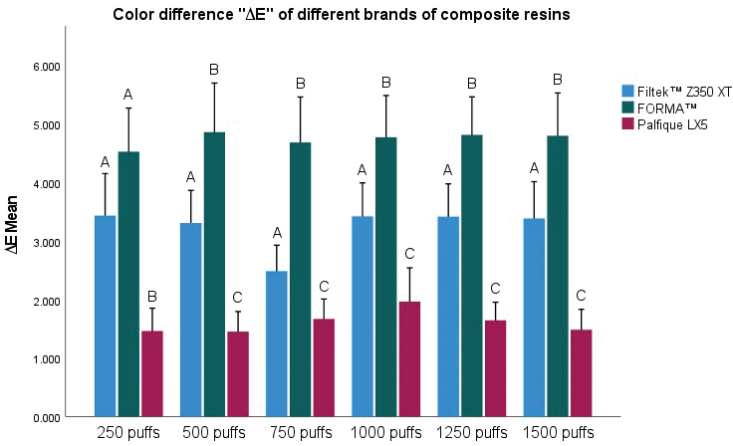
Colour difference ‘∆E’ of different brands of composite resins exposed to 250, 500, 750, 1,000, 1,250, and 1,500 puffs of electronic cigarette vapour. Bars represent mean ∆E values ± SD. Different letters indicate statistically significant differences between materials at the same exposure point (*p* < 0.05). SD: Standard Deviation.

Figure 3 shows the three brands of nanohybrid composite resins submitted to exposures to 250, 500, 750, 1,000, 1,250, and 1,500 puffs of e-cigarette vapour. At each measurement point, Filtek™ Z350 XT exhibited changes in colour, with ∆E values ranging from 2.485 ± 0.995 to 3.431 ± 1.618, while FORMA™ revealed the most pronounced alterations between 4.522 ± 1.677 and 4.854 ± 1.889, and Palfique LX5 showed the smallest colour shifts, ranging from 1.452 ± 0.776 to 1.964 ± 1.302. At each measurement point, Filtek™ Z350 XT exhibited colour changes, with ∆E values ranging from 2.485 ± 0.995 to 3.431 ± 1.618, while FORMA™ revealed the most pronounced alterations between 4.522 ± 1.677 and 4.854 ± 1.889, and Palfique LX5 showed the smallest colour changes, ranging from 1.452 ± 0.776 to 1.964 ± 1.302. Furthermore, the mixed ANOVA with Games-Howell post hoc test showed statistically significant differences at each puff interval (*p* < 0.05), as indicated by the different letters above the bars, which denote differences between materials at the same exposure point. In contrast, the p‑values associated with the intra-subject factor, which evaluated the variation of ∆E throughout the different puff intervals within each resin, showed values of 0.277 for Filtek™ Z350 XT, 0.978 for Forma™, and 0.303 for Palfique LX5, which indicated that no significant chromatic changes (*p* > 0.05) were observed in any material when the number of puffs increased, with their colour properties remaining relatively stable throughout the different exposure cycles. Detailed data on the mean chromatic difference (∆E), standard deviation and *p*-values for the materials are provided in Supplementary Table 1.

**Figure 4:**
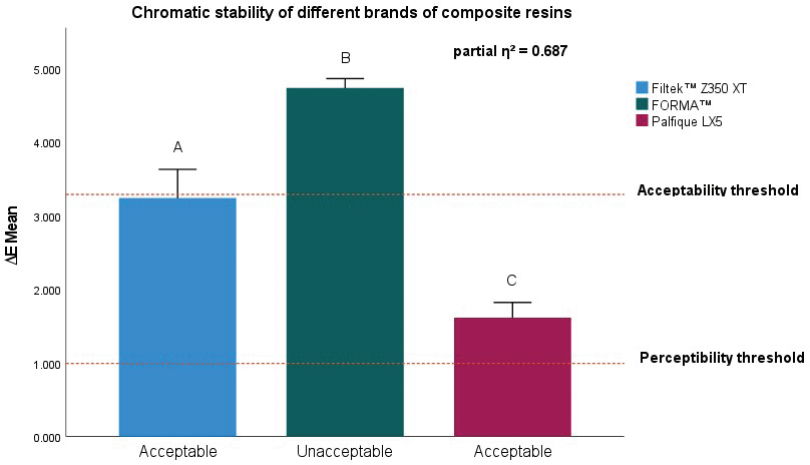
*In vitro* effect of electronic cigarette vapour on the chromatic stability of different brands of composite resins. Bars represent mean ∆E values ± SD. Different letters indicate statistically significant differences between materials (*p* < 0.05). The dashed lines represent the acceptability and perceptibility thresholds. SD: Standard Deviation.

Figure 4 summarises the *in vitro* effect of e-cigarette vapour on the chromatic stability of the three nanohybrid composite resins. Filtek™ Z350 XT demonstrated an overall ∆E value of 3.239 ± 0.284, which was just below the acceptability threshold and therefore clinically acceptable. In contrast, FORMA™ resin showed the highest colour change, with a ∆E of 4.737 ± 0.284, thus exceeding the acceptability threshold and considered clinically unacceptable, whereas Palfique LX5 presented a ∆E of 1.614 ± 0.284, within the acceptable range. None of the resins were positioned below the perceptibility threshold. The mixed ANOVA showed highly significant intersubject differences between the three materials (*p* < 0.05), with a large effect size (partial η² = 0.687), indicating that 68.7% of the variability in ∆E was attributable to the resin brand. Furthermore, the Games-Howell post hoc test confirmed statistically significant pairwise differences, as shown by the different letters above the bars. Detailed data are provided in Supplementary Table 2.

## Discussion

The results obtained demonstrated that exposure to e-cigarette vapour generated significant chromatic differences in the composite resins, with Palfique LX5 being the most stable and FORMA™ the most susceptible to colour change. With respect to FORMA™ and Filtek™ Z350 XT resins, their results were in agreement with the data reported by Vohra et al. [21] who showed that when they evaluated the effect of electronic cigarette vapour on nanohybrid resin discs, they found clinically perceptible and unacceptable chromatic differences between them. In a similar manner, Mood et al. [3], evaluated the chromatic stability of two brands of nanohybrid resin against electronic when they were exposed to electronic cigarettes, and they found clinically unacceptable values after this type of exposure. In contrast, Soliman et al. [7], showed that flavoured e-cigarette aerosol with a high concentration of nicotine produced clinically unacceptable colour changes in other aesthetic restorative materials such as ceramic veneers. Furthermore, Alrabeah et al. [4] found that several ceramic materials exhibited significant chromatic alterations when exposed to vaping devices.

These chromatic changes could be explained by physical-chemical mechanisms that affect both the organic matrix and the interface in inorganic fillers [22, 23]. Cigarette vapour contains compounds such as glycerine, propylene glycol, nicotine, and traces of aldehydes and metals, which can penetrate the resin surface and cause adsorption and absorption of these agents in the polymer matrix [22, 24, 25]. This interaction facilitates the incorporation of chromophores and the alteration of light refraction on the surface and subsurface of the material, thereby generating perceptible changes in colour [23, 26].

To ensure experimental reproducibility and control of the exposure variables, a protocol was established based on a defined number of puffs and daily cycles, avoiding replicating the continuous usage pattern characteristic of electronic cigarettes. This methodological decision responds to the high variability in actual consumption, as devices can hold up to 1,500 puffs, and their duration depends on the level of nicotine dependence and frequency of use, which can extend from a few days to several weeks or months [27, 28]. Recent studies have reported that regular electronic cigarette users consume between 135 and 250 puffs per day, although in cases of intensive use, this number can be higher [29]. Therefore, a controlled exposure was chosen that adequately represents the cumulative impact of vaping, without compromising the integrity of the samples or introducing biases derived from individual variability. This methodological strategy aligns with previous research in dental toxicology and bioaerosol analysis, where experimental consistency is prioritised over a complete simulation of user behaviour [30].

The chromatic changes observed across the tested resins showed a marked colour difference (ΔE) from approximately 250 puffs; however, additional exposures did not produce appreciable increases, suggesting colour stabilisation. This behaviour can be explained by the staining kinetics, in which rapid adsorption and absorption of chromophores into the superficial layers lead to early saturation. Assaf et al. [31] reported an initial increase in ΔE followed by stabilisation; Paolone et al. [32] described staining curves approaching an asymptote after repeated exposures; and Cinelli et al. [33] showed that further penetration depends on the matrix and filler size, so once the accessible regions are saturated diffusion into the bulk is slow. These mechanisms account for the pronounced early alteration and the lack of significant changes in subsequent exposure cycles.

In this investigation, the high-loading nanohybrid resin based on a Bis-GMA (bisphenol A glycidyl methacrylate) and UDMA (urethane dimethacrylate) matrix, such as Filtek™ Z350 XT, showed an initial reduction of L* followed by a partial recovery, and was accompanied by a sustained increase in the b* parameter which indicated a yellowing effect. Although its ΔE values remained within the clinically acceptable threshold (≤ 3.3) up to 750 puffs, values from 1,000 puffs onwards revealed clinically perceptible changes. These findings were explained by the presence of their high surface area due to the size of the nanoparticles, which favours the absorption of pigments, if the procedure is not accompanied by optimal polishing. Christiani et al. [34], have previously demonstrated this behaviour in chromogenic media such as coffee and wine.

Moreover, these results were supported by the study conducted by Barbosa et al. [26] who evaluated the effect of conventional cigarette smoke on micro-hybrid and nanohybrid composites; they concluded that nanohybrid resins such as Filtek™ Z350 XT showed less colour change when compared with controlled exposure to smoke, however, with statistically significant differences as exposure increased. This supports the progressive trend towards colour change observed in the present study when samples were submitted to e-cigarette aerosol.

FORMA™ showed the highest ΔE values from the very first exposures and kept them above the clinical acceptability threshold (ΔE > 3.3) throughout all cycles, indicating a marked susceptibility to e-cigarette vapour. This greater susceptibility may be related to higher permeability of its resin matrix and/or a rougher finished surface, facilitating penetration of aerosol compounds and pigment retention [35]; consequently, its indication would be inadvisable in patients with frequent vaping habits. These observations also underscore the clinical relevance of perceptibility and acceptability ΔE thresholds when selecting restorative materials for people who vape. In this vein, additional microstructural or surface-roughness data before and after exposure could clarify the mechanisms underlying this behaviour [35, 36]. Alnasser et al. [12] also pointed out a similar situation, in which resins with a higher level of water absorption presented greater degree of pigment retention, thereby aggravating chromatic alterations.

Filtek™ Z350 XT resin showed intermediate ΔE values, some within the acceptable range and others slightly exceeding it, with variable behaviour throughout the increasing cycles of puffs. Although this resin had a certain initial chromatic resistance, its response to e-cigarette vapour was not completely stable and tended to fluctuate. Although it was not the most affected resin, it did not guarantee long-term aesthetic stability when submitted to this type of exposure. In contrast, Palfique LX5 resin demonstrated a superior performance; both relative to the L*, a*, b* values, and to total colour difference (ΔE) parameters. It showed stability and uniformity, with variations below the clinical acceptability threshold. This stability may have been due to its submicron-sized rate of filler system reinforced with silica and zirconium, in addition to a low rate of water absorption and lower reactivity to external agents. Precisely, studies such as those conducted by Soliman et al. [7] and Mahmoud et al. [8] have demonstrated that materials with a higher rate of conversion and low solubility perform better against vaping-derived colouring agents.

It is also important to point out the data mentioned by Vohra et al. [21], who established that electronic nicotine delivery devices generated aerosols with a complex chemical composition (including nicotine, propylene glycol, glycerine and aromatic compounds) that can induce oxidative, cytotoxic, and physical effects on dental materials. The cited study pointed out that resins exposed to these aerosols present effects similar to those observed with conventional cigarette smoke, especially with regard to surface deterioration and the loss of optical properties, therefore, the effects of vaporisers should not be underestimated.

Continuous exposure to e-cigarettes can compromise the aesthetic longevity of restorative materials and thus impact various oral health problems or even patients’ quality of life [5, 32, 37]. Therefore, it is essential to consider patient habits when planning aesthetic restorative treatments, in order to select the most appropriate material for each case, thereby promoting evidence-based decision-making.

Among the limitations of this study is it’s *in vitro* design. While this allowed for the control of variables, it did not fully represent real intraoral conditions, such as salivary flow, friction, or pH variability. Furthermore, only three brands of composite resin and a single e-cigarette formulation were evaluated, limiting the generalisability of the results. Exposure to aerosol was standardised, but it did not take into account the diversity of vaping habits among users. In addition, it was not possible to measure the exact volume dispensed in each puff; however, it should be noted that other studies also did not include this analysis, although it would be advisable to incorporate it in future research to ensure a more precise dosage. The findings of this work also highlight the need for more comprehensive future analyses, including the evaluation of resin surface and microstructure, the use of scanning electron microscopy (SEM) to observe changes before and after exposure, the testing of different e-liquid formulations, and long-term simulations that incorporate more clinically realistic conditions such as brushing cycles and salivary immersion. In this regard, the generation of more representative studies building upon this type of research may contribute to more informed decision-making regarding the choice of resin, particularly for patients with vaping habits.

## Conclusions

Based on the findings of this study, it is concluded that when the colour difference ‘ΔE’ values obtained were compared after progressive exposure to electronic cigarette vapour, FORMA™ resin was the most affected, since it showed clinically unacceptable colour variation; whereas Filtek™ Z350 XT and Palfique LX5 resins showed clinically acceptable colour variation values. Palfique LX5 had the greatest resistance to colour change and therefore had the best chromatic stability.

## Declaration concerning interest

The authors report that there is no conflict of interest to declare.

## Declaration of authors’ contributions

AVAV: Conceptualization; research; original draft; methodology; validation; writing, review, and editing; data curation. YFCV: Conceptualization; research; methodology; writing, review, and editing; formal analysis; supervision and resources. TVUC: Conceptualization and resources; writing, review, editing, and supervision. CXPC: Conceptualization and resources; writing of the original draft; writing, review, editing, and supervision.

## Declaration about availability of data

Data can be shared upon request.

## Supplementary Material


