# Supplementary material for: Influence of electronic cigarette vapour on the in vitro chromatic stability of different nanohybrid composite resins
Source: Biomater Investig Dent. 2025 Oct 13;12:44849. doi: 10.2340/biid.v12.44849 (PMC12536651; doi:10.2340/biid.v12.44849)
Supplement: Supplementary file 1 [file BIiD-12-44849-s1.pdf]

Supplementary material has been published as submitted. It has not been copyedited or typeset by Biomaterial Investigations in Dentistry.

Supplementary Tables

Supplementary Table 1. Color difference "ΔE" of different brands of composite resins exposed to 250, 500, 750, 100, 1250, 1500 puffs of electronic cigarette vapor.

Electronic cigarette vapor	Filtek™ Z350 XT	FORMA™	Palfique LX5	*p-value**
	ΔE Mean (SD)	ΔE Mean (SD)	ΔE Mean (SD)	
**250 puffs**	3.431 (1.618) ^{A}	4.522 (1.677) ^{A}	1.464 (0.878) ^{B}	<0.001**
**500 puffs**	3.306 (1.250) ^{A}	4.854 (1.889) ^{B}	1.452 (0.776) ^{C}	<0.001**
**750 puffs**	2.485 (0.995) ^{A}	4.678 (1.751) ^{B}	1.670 (0.764) ^{C}	<0.001**
**1000 puffs**	3.417 (1.292) ^{A}	4.767 (1.607) ^{B}	1.964 (1.302) ^{C}	<0.001**
**1250 puffs**	3.415 (1.260) ^{A}	4.808 (1.461) ^{B}	1.647 (0.696) ^{C}	<0.001**
**1500 puffs**	3.381 (1.420) ^{A}	4.793 (1.648) ^{B}	1.485 (0.788) ^{C}	<0.001**
p-value*	0.277	0.978	0.303	

ΔE: Color difference; SD: Standard Deviation; *Significance test of the Between-subject Factor (Nanohybrid resins) and Within-subject Factor (E-cigarette vapor puffs) of the mixed ANOVA.

**There are statistical differences between the groups (p<0.05)

Note: The Games-Howell post hoc test was performed for multiple comparisons of the intersubject factor, the different superscript letters between the groups indicate that there are statistically significant differences between them (p<0.05)

Supplementary Table 2. *In vitro* effect of electronic cigarette vapor on the chromatic stability of different brands of composite resins.

	Filtek™ Z350 XT	FORMA™	Palfique LX5	*p-value** / partial η^2
	ΔE Mean (SD)	ΔE Mean (SD)	ΔE Mean (SD)	
**Color difference**	3.239 (1.306) ^{A}	4.737 (1.672) ^{B}	1.614 (0.867) ^{C}	<0.001**
**Chromatic stability**	Acceptable	Unacceptable	Acceptable	0.687

ΔE: Color difference; SD: Standard Deviation; partial η^2: Effect size; *Significance test of the Intersubject Factor (Nanohybrid resins) of the mixed ANOVA

**There are statistical differences between the groups (p<0.05)

Note: The Games-Howell post hoc test was performed for multiple comparisons of the intersubject factor, the different superscript letters between the groups indicate that there are statistically significant differences between them ($p<0.05$)
